# DeepSAHF: deep learning-based detection of senescence-associated chromatin features reveals nuclear heterogeneity

**DOI:** 10.1093/bib/bbaf631.040

**Published:** 2025-12-12

**Authors:** Claire Yik-Lok Chung, Shinya Ohta, Hideki Tanizawa, Ken-ichi Noma

**Affiliations:** Institute for Genetic Medicine, Division of Genome Biology, Hokkaido University; Institute for Genetic Medicine, Division of Genome Biology, Hokkaido University; Institute for Genetic Medicine, Division of Genome Biology, Hokkaido University; Institute for Genetic Medicine, Division of Genome Biology, Hokkaido University; Department of Biology, Institute of Molecular Biology, University of Oregon

## Abstract

Senescence-associated heterochromatin foci (SAHF) are condensed chromatin domains that mark cellular aging. Manual microscopy-based counting of SAHF-positive nuclei is widely used but is labor-intensive, subjective, and limited by binary classification that overlooks nuclear heterogeneity, especially in intermediate senescence or under non-canonical chromatin features. Accurate automated detection remains challenging due to variability in nuclear morphology, fluorescence intensity, and imaging conditions, with current approaches relying on arbitrary thresholds that hinder reproducibility.

We present DeepSAHF, a deep learning–based pipeline for robust and reproducible detection of senescence-specific chromatin states from fluorescence microscopy images. The workflow integrates image pre-processing, YOLO-based single nucleus segmentation, and chromatin pattern classification to identify SAHF-positive cells. An extension using a visual language model (VLM) systematically comments on nuclear shape and features, facilitating interpretability and filtering unsuitable nuclei (e.g., out-of-focus). DeepSAHF is trained and tested at 20× resolution on oncogene-induced senescence cells across multiple time points, using curated high-confidence images from literature and novel data to ensure generalization without manual thresholds.

By enabling scalable, reproducible, and interpretable analysis of chromatin reorganization, DeepSAHF advances beyond traditional methods, opening avenues to explore nuclear heterogeneity in aging and stress responses, with broad applications in drug screening, regenerative medicine, and cancer biology.

